# Evaluation of Simplified HCV Diagnostics in HIV/HCV Co-Infected Patients in Myanmar

**DOI:** 10.3390/v15020521

**Published:** 2023-02-13

**Authors:** Phyo Pyae Nyein, Shane Tillakeratne, Sabai Phyu, Myint Myint Yee, Mya Mya Lwin, Kyaw Linn Htike, May Thu Aung, Jason Grebely, Tanya Applegate, Josh Hanson, Gail Matthews, Kyaw Swar Lin

**Affiliations:** 1 Specialist Hospital Mingaladon, Yangon X42H+J4, Myanmar; 2 The Kirby Institute, University of New South Wales, Sydney, NSW 2052, Australia; 3 Department of Tropical and Infectious Diseases, Specialist Hospital Waibargi, Yangon W5C4+6J7, Myanmar; 4 Department of Microbiology, University of Medicine 2, Yangon 644-704, Myanmar; 5 Myanmar-Australia Research Collaboration for Health Laboratory, Yangon W5C4+6J7, Myanmar; 6 Cairns and Hinterland Hospital and Health Service, Cairns North, QLD 4870, Australia

**Keywords:** hepatitis C, DAA, hub-and-spoke, POC, LMIC, Myanmar

## Abstract

To evaluate a decentralised testing model and simplified treatment protocol of hepatitis C virus (HCV) infection to facilitate treatment scale-up in Myanmar, this prospective, observational study recruited HIV–HCV co-infected outpatients receiving sofosbuvir/daclatasvir in Yangon, Myanmar. The study examined the outcomes and factors associated with a sustained virological response (SVR). A decentralised “hub-and-spoke” testing model was evaluated where fingerstick capillary specimens were transported by taxi and processed centrally. The performance of the Xpert HCV VL Fingerstick Assay in detecting HCV RNA was compared to the local standard of care ( plasma HCV RNA collected by venepuncture). Between January 2019 and February 2020, 162 HCV RNA-positive individuals were identified; 154/162 (95%) initiated treatment, and 128/154 (84%) returned for their SVR12 visit. A SVR was achieved in 119/154 (77%) participants in the intent-to-treat population and 119/128 (93%) participants in the modified-intent-to-treat population. Individuals receiving an antiretroviral therapy were more likely to achieve a SVR (with an odds ratio (OR) of 7.16, 95% CI 1.03–49.50), while those with cirrhosis were less likely (OR: 0.26, 95% CI 0.07–0.88). The sensitivity of the Xpert HCV VL Fingerstick Assay was 99.4% (95% CI 96.7–100.0), and the specificity was 99.2% (95% CI 95.9–99.9). A simplified treatment protocol using a hub-and-spoke testing model of fingerstick capillary specimens can achieve an SVR rate in LMIC comparable to well-resourced high-income settings.

## 1. Introduction

The introduction of direct acting antiviral (DAA) regimens has revolutionised the management of hepatitis C virus (HCV) infection. The high efficacy and low toxicity of these regimens mean that on-treatment virological monitoring is no longer required and that the monitoring for toxicity is minimised [1]. DAA therapy has improved HCV management in low- and middle-income countries (LMIC), which have the highest global HCV burden [2]. Although Myanmar has set national targets to diagnose and treat 50% of people living with HCV by 2030, the current HCV testing and treatment levels are inadequate [3]. The cost of diagnostics and DAA therapy [4], and the accessibility to testing and treatment remain barriers to HCV treatment in Myanmar [5], similar to other LMIC [6,7]. 

In Myanmar, the prevalence of HCV infection is estimated to be 2.7%; this equates to at least 1.1 million people living with chronic HCV [8]. In people living with Human Immunodeficiency Virus, estimates of HCV co-infection range from 5% to 23% [5,9]. Without a treatment scale-up, it is estimated that 333,000 new HCV infections and 97,000 HCV-related deaths will occur in Myanmar between 2020 and 2030 [3]. Standard of care HCV RNA testing is available throughout Myanmar as part of the National Hepatitis Control program that was launched in 2017, and the national HCV treatment guidelines—issued in the same year—provide a comprehensive treatment pathway: antibody screening by immunoassay, a confirmation by viral load testing, a series of pre-treatment assessments ( HIV testing, liver staging and renal function) and a confirmation of SVR 12 weeks post-treatment [10]. While the program provided testing and treatment for more than 8000 people from 2017–2020 [11], national data on the SVR rates are not available. Furthermore, the overall time between the standard of care HCV RNA testing, pre-treatment assessments, DAA initiation and laboratory confirmation of a cure can vary across the provinces of Myanmar, as accessibility to testing and treatment is limited [5]. Public sector treatment is only possible in secondary and tertiary hospitals and is informed by testing that is performed at reference laboratories in Yangon and Mandalay, Myanmar’s largest cities [3]. Simplification and decentralisation of the current HCV management algorithms are crucial for a treatment scale-up.

Point-of-care (POC) HCV RNA testing with the Xpert HCV VL Fingerstick test has a good diagnostic accuracy compared to laboratory-based testing following a venepuncture sampling [12,13]. POC HCV RNA testing reduces the transport and laboratory costs [14,15] and it shortens the time to treatment initiation [16,17,18]. While molecular POC testing has been implemented in some resource-limited settings, few studies have evaluated its efficacy. Furthermore, POC molecular testing may not be viable in resource-limited settings due to the cost and accessibility, particularly in rural and remote regions [19,20]. 

Decentralised hub-and-spoke models where POC fingerstick specimens are collected at clinical sites (spokes), and then transported using a fingerstick EDTA collection tube to the nearest GeneXpert machine, possibly located some distance away (i.e., a hub), may be more successful. This innovative model has been demonstrated for other infectious diseases [21,22,23], but not for HCV. 

This prospective, observational study evaluated virological outcomes using a simplified model of care in HIV–HCV co-infected individuals undergoing DAA therapy in hospital outpatient settings in Yangon, Myanmar. This study compared the sensitivity and specificity of the Xpert HCV Viral Load VL Fingerstick Assay for HCV RNA detection (i.e., fingerstick capillary whole blood samples transferred in a microvette EDTA collection tube), to the Xpert HCV Viral Load assay (plasma collected by venepuncture) before and following the DAA therapy in the context of the novel hub-and-spoke model.

## 2. Materials and Methods

### 2.1. Study Design and Participants

From 22 January 2019 to 3 February 2020, the participants were screened in the HIV outpatient departments of the Specialist Hospital Mingaladon and Specialist Hospital Waibargi. All the enrolled participants were HIV-1 antibody-positive and HCV antibody-positive. The national guidelines for simplified HCV treatment were used to determine treatment eligibility [10]. Individuals who were unwilling or unable to provide informed consent, had an impaired renal function, were pregnant, were taking prohibited concomitant medication, or had a significant illness that would interfere with the treatment, assessment, or adherence to the study protocol were excluded.

### 2.2. Study Assessments

Study assessments were conducted at two on-site visits: pre-treatment screening and 12 weeks post-treatment (SVR12). The participants completed an interviewer-administered questionnaire on a tablet computer at both visits, collecting their demographic data, history of drug/alcohol use, and previous incarceration. A standardised measure of the quality of life (EQ-5D-5L), including a visual analogue scale (VAS) measuring the overall perceived health status (out of 100), was collected (Table S1) [24]. Current alcohol consumption was evaluated using the Hazard Alcohol Use Identification Test (AUDIT-c), with a hazardous consumption indicated by scores of >3 (for women) and >4 (for men) [25]. 

Within one week of screening, participants with detectable HCV RNA (>10 IU/mL) were offered sofosbuvir + dose-adjusted daclatasvir therapy (without cirrhosis—12 weeks therapy, and with cirrhosis—24 weeks therapy). During treatment, the participants attended the outpatient department monthly to collect their medication. A post-treatment clinical assessment was performed for the participants who returned for their SVR12 visit.

### 2.3. Diagnostic Testing Methodology

HIV Antibody testing: Confirmation of the HIV status was performed in all participants using three approved rapid HIV antibody tests: Determine^®^ HIV-1/2 (Abbott, Chicago, IL, USA), Uni-Gold™ HIV-1/2 (Trinity Biotech, Co Wicklow, Ireland), and HIV1/2 Stat-Pak Dipstick (Chembio Diagnostic System, Inc, Medford, NY, USA) [26]. 

HCV Antibody testing: A positive HCV antibody was confirmed in all the participants at screening using a World Health Organization (WHO) prequalified POC HCV antibody test (SD, Abbott, Chicago, IL, USA) [26]. 

Standard of care HCV RNA testing: The presence of HCV RNA in plasma (derived from 6mL whole-blood) collected by standard venepuncture was determined at both scheduled visits as per the national Myanmar HCV treatment guidelines (Myanmar Ministry of Health and Sports; 2019), using an Xpert HCV Viral Load assay on the GeneXpert R2 6-colour, 4 module machine (GXIV-4-L System, 900-0513, GeneXpert Dx software v4.6a, lower limit of quantification: (LLoQ) 10 IU/mL; Cepheid, Sunnyvale, CA, USA) [27]. HCV RNA genotyping is not a prerequisite for HCV treatment in Myanmar and was not performed.

Fingerstick HCV RNA testing: A fingerstick capillary whole-blood sample (100 µL) was collected into a microvette collection tube (Microvette^®^ 100 μL K3EDTA, Sarstedt, Nümbrecht, Germany) at both visits using a MiniCollect^®^ Safety Lancet (Greiner Bio-One, Kremsmünster, Austria), as per the manufacturer’s directions and WHO guidelines [28]. The samples were transported at ambient temperature by a same-day taxi (or motorbike when a taxi was not available) to the Myanmar–Australia Research Collaboration for Health (MARCH) laboratory in Yangon. The transit time between the collection and delivery of the samples ranged between 15 and 30 min. The majority (87%) of the POC HCV RNA testing was performed on the same day of the sample collection, with the remainder of the samples tested 1–4 days later. The HCV RNA testing was performed using the Xpert HCV VL Fingerstick Assay (Cepheid, Sunnyvale, CA, USA; (LLOQ): 100 IU/mL, upper limit of quantification: 10^8^ log_10_ IU/mL; 100% sensitivity, 100% specificity) on a GeneXpert R2 6-colour, 2 module machine (GXII-2-L System, GeneXpert Dx software v4.6a; Cepheid, Sunnyvale, CA, USA) (Cepheid, 2019). The GeneXpert enables the quantification of HCV RNA through real-time PCR technology, with the time to result taking approximately one hour [29].

### 2.4. Study Definitions

Cirrhosis was defined clinically or using the aspartate aminotransferase to platelet ratio index (APRI) ≥ 2.0. 

Current injecting drug use (IDU) was defined as injecting in the last month. 

HCV virological suppression was defined as a HCV RNA below the lower limit of quantitation (LLoQ) (target not detected (TND), or target detected, not quantifiable (TD, nq)). 

Sustained Virologic Response (SVR) was defined as a HCV RNA target not detected (TND) or target detected, not quantifiable (TD, nq) at the post-treatment week 12. 

HCV virologic failure was defined as a non-response or failure of virological suppression with quantifiable HCV RNA at the end of treatment.

Invalid results from the Xpert HCV VL Fingerstick Assay occur when no HCV RNA result is obtainable as a result of samples not being properly processed, a reverse transcription PCR (RT-PCR) inhibition is encountered due to sample integrity, or the sample is improperly collected and transported [29].

Error results from the Xpert HCV VL Fingerstick Assay occur when no HCV RNA result is available and the GeneXpert encounters a system component failure, a reagent check fails, or a PCR probe check fail that terminates the assay [29].

First-pass testing was defined as testing performed without the repeat testing of invalid or error results.

### 2.5. Study Endpoints

The primary endpoint of the study was SVR12 (undetectable HCV RNA at >12 weeks following the treatment completion) by an intention-to-treat (ITT) analysis (for all participants who received ≥1 dose of the study drug). A modified intention-to-treat (mITT) analysis was also conducted, including only those patients with available SVR12 HCV RNA results. A secondary analysis was performed which evaluated the sensitivity and specificity of the Xpert HCV VL Fingerstick Assay using capillary blood collected via fingerstick testing, and compared it with the standard of care plasma HCV RNA collected via venepuncture (Xpert HCV Viral Load Assay).

### 2.6. Statistical Analysis 

The data were collected electronically and analysed using statistical software (STATA version 14.0; Stata Corporation, College Station, TX, USA). The proportion of the participants achieving SVR confirmed through the standard of care HCV RNA testing was calculated in both the ITT and mITT populations. 

Logistic regression analysis was used to identify the factors associated with SVR (in the ITT and mITT populations). All variables were considered in multivariate logistic regression models using a Firth-type logistic regression to reduce small sample biases [30]. The final models included factors remaining significant at the 0.05 level.

The sensitivity and specificity of HCV RNA on the GeneXpert machine using capillary blood collected via fingerstick was compared using the standard of care HCV RNA plasma testing as the gold standard. This included both positive and negative samples at screening, following the completion of treatment among the participants who were enrolled in this study. Any discordant results were included in all calculations of the sensitivity and specificity. Bland–Altman difference plots were generated to assess the bias and agreement measurements, including the limits of agreement between the quantification of HCV by the Xpert HCV VL Fingerstick Assay compared to the standard of care. The data were analysed with GraphPad Prism (version 7.03).

## 3. Results

### 3.1. Participant Characteristics 

**Figure 1:**
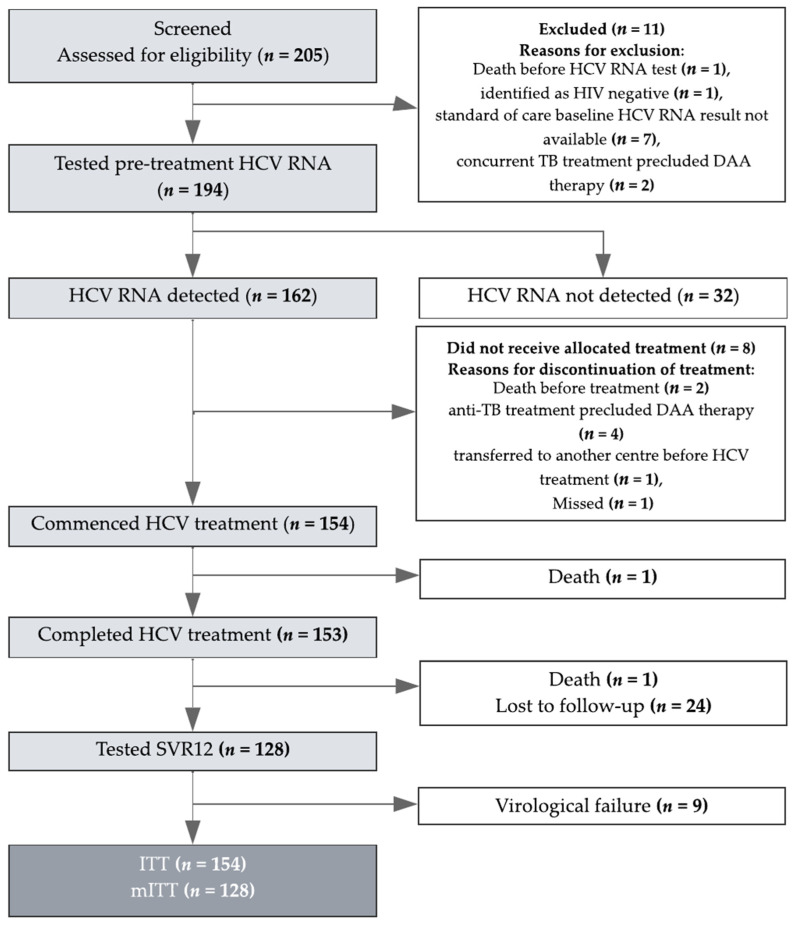
Patient Disposition. HCV: hepatitis C virus; ITT: intention-to-treat; mITT: modified intention-to-treat. The number of participants screened (194/205; 95%), with detectable HCV RNA (162/194; 84%) and who commenced treatment (154/162; 95%) is depicted. Of the 11 patients who were excluded at screening, 7/11 (64%) did not have a standard of care HCV RNA result available at the time of treatment assessment, 2/11 (18%) had concurrent treatment, 1/11 (9%) died prior to HCV RNA testing, and 1/11 (9%) was identified as HIV-negative. Among those 8/162 (5%) who did not commence treatment, 2/8 (25%) died before treatment, 4/8 (50%) were receiving anti-TB treatment precluding DAA therapy, 1 was transferred to another centre before treatment and 1 missed the treatment. One death occurred during treatment due to hepatic decompensation. This participant had underlying cirrhosis and died 15 weeks after commencing therapy.

Of the 205 participants screened, 194/205 (95%) were tested for HCV RNA, of whom 162 (84%) were HCV RNA-positive (Figure 1).

The median (interquartile (IQR)) age was 39 (29–47) years, 54/194 (28%) were female, and 165/194 (85%) were receiving antiretroviral therapy (ART), predominantly tenofovir, lamivudine and efavirenz combination therapy (Table 1). 

At screening, the CD4 cell count was ≥200 cells/µL in 129/194 (67%) and <100 cells/µL in 26/194 (14%) (Table 1). The median (IQR) HCV RNA viral load was 6.2 log_10_ (5.4–6.6) IU/mL. Cirrhosis was present in 15/194 (8%). The median (IQR) score for the self-reported perception of health was 85 (80–90) (Table S1).

### 3.2. Treatment Initiation, Uptake, and Completion

Of the 162 participants with detectable HCV RNA at screening, 154 (95%) initiated treatment and 153 (99%) completed treatment (Figure 1). Of these 153, 128 (84%) returned for their SVR12 visit and were tested for HCV RNA. Of the 24/153 (16%) lost to follow-up, 18/24 (75%) occurred in 2020 after the onset of the severe acute respiratory syndrome coronavirus 2 (SARS-CoV-2) pandemic and the resulting stay-at-home orders in Yangon. One death occurred during treatment due to hepatic decompensation. This participant had underlying cirrhosis and died 15 weeks after commencing therapy.

### 3.3. Treatment Efficacy and Factors Associated with SVR

**Figure 2:**
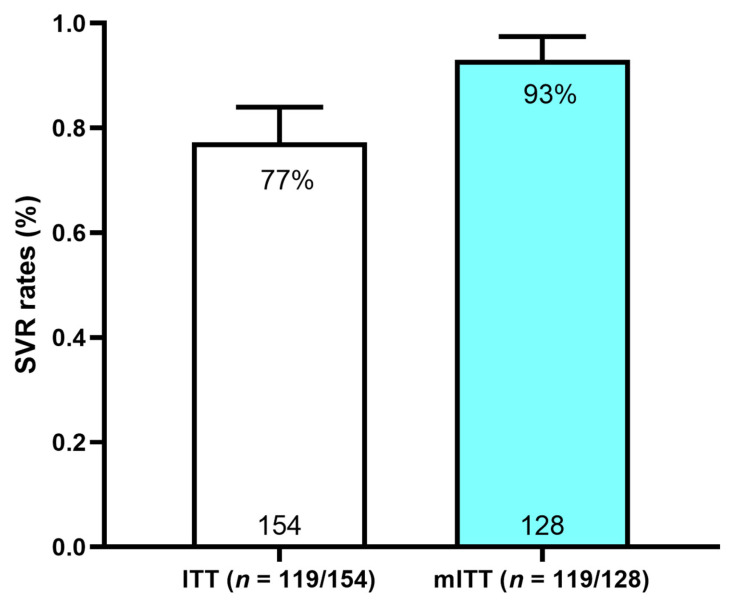
SVR12 rates in ITT and mITT populations. SVR: sustained virologic response; ITT: intention-to-treat; mITT: modified intention-to-treat.

A SVR was achieved in 119/154 (77%) in the ITT and 119/128 (93%) in the mITT populations (Figure 2).

Baseline characteristics that were associated with the SVR are presented in Table 2 (for the ITT and mITT populations). In a multivariate analysis for the ITT population, the employment (with an odds ratio (OR): 2.79, 95% confidence interval (CI): (1.15–6.79), *p* = 0.02) and APRI ≥ 2 (OR 0.26, (0.07–0.88), *p* = 0.03) were independently associated with SVR. 

In the multivariate analysis for the mITT population, only the receipt of ART was independently associated with SVR (OR 7.16 (95% CI: 1.03–49.50) *p* = 0.04).

### 3.4. Virological Failure

Virological failure was observed in 9/128 (7%) participants (Table 3).

All nine of these participants had no evidence of cirrhosis and a CD4 ≥ 200 cells/µL, but only 4/9 (44%) were on ART. The median (IQR) baseline for the HCV RNA viral load was 6.4 (5.8–6.6) log_10_ IU/mL. 

### 3.5. Diagnostic Performance of Fingerstick Testing

Of the 205 participants, 194 had available samples for testing (Figure S1). In total, 343 Xpert HCV VL Fingerstick Assay test runs were performed during the study; however, 35/343 (10%) resulted in an invalid result reading, and 9/343 (3%) had an error result reading. Where possible, subsequent testing on a recollected sample was attempted resulting in an additional 27 (27/44; 61%) valid results, and 4 (4/44; 9%) error and 13 (13/44; 30%) invalid result tests that were unable to be retested (Figure S1). There was a total of 299 instances where both a valid Xpert HCV VL Fingerstick test and a standard of care HCV RNA test result were available.

### 3.6. Sensitivity & Specificity Analysis

Among the samples with a valid test result, the sensitivity of the Xpert HCV VL Fingerstick Assay for HCV RNA detection in the samples collected by fingerstick capillary whole blood was 99.4% (95% CI 96.7–100.0), and the specificity was 99.2% (95% CI 95.9–99.9; Table 4). The positivity predictive value (PPV) was 99.3% (95% CI 95.8–99.9%) and negative predictive value (NPV) was 99.2% (95.0–99.8%). As the study was conducted in a clinical setting, samples that produced an invalid or error result were recollected where possible, and a sensitivity and specificity analysis was also performed as a first-pass (testing performed without the repeat testing of invalid or error results) to compare the estimates (Table S2).

**Figure 3:**
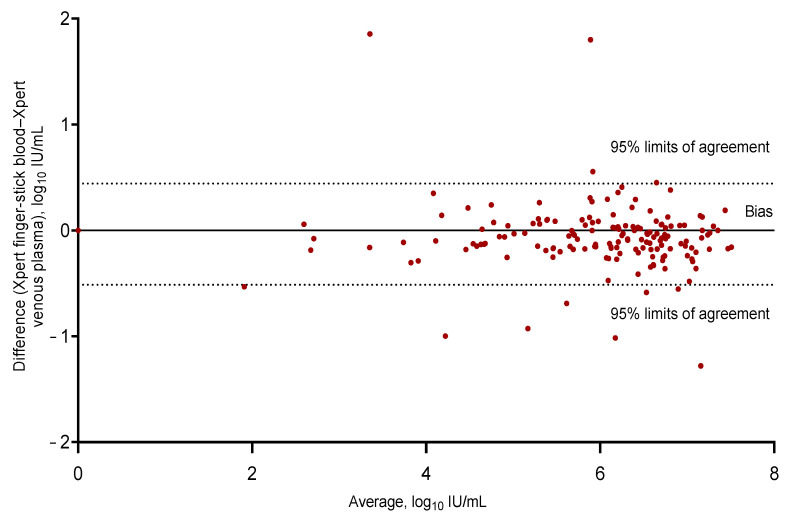
Bland–Altman bias plot of differences for the Xpert HCV VL Fingerstick Assay for HCV RNA detection in fingerstick capillary whole-blood samples compared with the Xpert HCV Viral Load Assay for HCV RNA detection in venous plasma (with two discrepant results excluded from the analysis); *n* = 297, bias −0.03643, 95% limits of agreement −0.51 to 0.44. HCV: hepatitis C virus.

As shown by the Bland–Altman plot analysis (Figure 3), the HCV RNA concentrations detected by the Xpert HCV Viral Load Assay using fingerstick capillary whole blood were a mean 0.03 (SD 0.24) log_10_ IU/mL lower than those measured by the standard of care, with 95% of the differences between −0.51 and 0.44 log_10_ IU/mL. Two screening visit samples had discordant results for the HCV RNA quantification with fingerstick capillary whole blood and were excluded in the Bland–Altman plot. In the first sample with a discordant result, the HCV RNA concentration was 7.6 log_10_ IU/mL when tested by the Xpert HCV VL Fingerstick Assay and was undetectable when tested by the standard of care. An Xpert HCV VL Fingerstick result confirmation was not performed due to an insufficient sample; however, confirmation testing by the standard of care was performed, and this participant subsequently did not receive treatment. In the second sample with a discordant result, the HCV RNA concentration was undetectable when tested by the Xpert HCV VL Fingerstick Assay and 3.7 log_10_ IU/mL when tested by the standard of care. As per the standard of care, this participant was initiated onto treatment.

## 4. Discussion

This study performed amongst HIV/HCV co-infected participants at two outpatient clinics in Yangon, Myanmar, demonstrates that participants managed using a simplified DAA treatment protocol can achieve excellent SVR rates, comparable to those achieved in resource-rich settings. It also highlights the excellent sensitivity and specificity of the Xpert HCV VL Fingerstick Assay for HCV RNA detection, suggesting that it may have a role in resource-limited settings in optimising access to care. Finally, the hub-and-spoke testing model—using a fingerstick EDTA collection tube—facilitated the remote sample collection with centralised testing. This provided the clinicians and patients with prompt clinical information, expediting the initiation of HCV therapy. The SVR of 93% in the mITT population is consistent with trials among HIV/HCV co-infected participants receiving DAA therapy in well-resourced settings [31,32,33]. This is particularly notable given the use of a first-generation regimen (i.e., sofosbuvir/daclatasvir) which is more complicated to administer and has greater drug–drug interactions than the newer pan-genotypic regimens. 

Employment was associated with SVR, suggesting a better treatment access and increased adherence in these individuals [34]. Cirrhosis was associated with a lack of SVR, further highlighting that these individuals are more difficult to cure [35]. ART was associated with SVR, suggesting a higher health engagement in individuals on ART and/or increased health provision for this population. Notably, in the participants with virological failure, less than half of the individuals were on ART, which might be explained by a greater geographical distance from health services, the presence of social stressors affecting the access to care, or poorer health literacy [36]. 

Although the SVR was lower in the ITT analysis (Figure 2), 75% of the participants that were lost to follow-up were unable to attend their SVR12 visit due to their expected clinic visit coinciding with a city-wide lockdown in Yangon in response to the SARS-CoV2 pandemic [37,38]. The participants were unable to access hospital services during the follow-up period, and clinicians were unable to transport specimens into Yangon for testing. Similar rates of loss to follow-up in 2020 have been reported in several studies [39,40,41]. Many of these participants completed their full course of therapy as directed, and most would, therefore, be anticipated to have achieved a cure. The inclusion of interventions designed to enhance the HCV follow-up are clearly vital in vulnerable populations with a high risk of HCV infection [42], to mitigate the loss to follow-up of patients especially during the ongoing global SARS-CoV2 pandemic [41]. 

The high sensitivity and specificity of the Xpert HCV VL Fingerstick Assay for HCV RNA detection by fingerstick with the use of a novel collection vessel is an important finding of this study. The Xpert HCV VL Fingerstick Assay demonstrated a strong agreement with the local standard of care assay (Xpert HCV VL Assay via venous plasma) with a 0.3 log_10_ IU/mL or lower difference between 95% limits of agreement of all the measurements across all the HCV RNA concentrations tested. High sensitivity and specificity estimates were also observed when the analysis was performed as a first-pass testing (Table S2). Although there were two discrepant results when comparing the Xpert HCV VL Fingerstick Assay for HCV RNA detection by fingerstick with the standard of care, these discrepancies at the screening visit were likely to be explained by human error and would be less likely to occur with more experience and improved quality control processes. 

Repeat testing of 44 (13%) fingerstick tests were required due to invalid and error results on the first-pass testing and are likely in part explained by logistical and environmental factors experienced during the study. The invalid results on the GeneXpert can be attributed to the sample condition level prior to testing (e.g., problems during the collection, transport, or sample environment). A small number of samples that missed taxi transport were transported by motorbike which may have resulted in conditions that may have affected the sample integrity. While the sample testing was performed on the same day of collection, there was a delay in testing in some samples (13% of all samples tested; range of 1–4 days post-sample collection), which may also have adversely affected sample quality. The effect of lag times between the blood draw and HCV RNA testing on the sample integrity has been reported previously [43]. Additionally, while the samples were transported at ambient temperature, the impact of the Yangon’s tropical climate meant that these temperatures approached 40 °C at some times and this may also have impacted the sample integrity. Our study has highlighted the potential for the hub-and-spoke model but also the requirement for temperature and transport safeguards to be firmly established to minimize retesting. 

HCV POC tests that resulted in an error on the GeneXpert can be attributed to machine or user errors encountered during the loading and testing of the sample and were low in our study (3%) and decreased in frequency as the study progressed, thus highlighting the importance of operator ongoing education, training, and competency assessment. A learning curve for laboratory scientists and clinicians can be expected when implementing new models of care and novel molecular techniques [44].

The hub-and-spoke model of testing with the GeneXpert platform with an EDTA microvette sample collection is novel. Decentralisation of the specimen collection and testing at POC is increasingly recognised in many infectious diseases as an ideal way to ensure prompt access to treatment [45], particularly in remote or difficult to access locations [46,47,48]. This model is particularly suited to infections where a diagnosis can be made immediately (through a lateral flow assay or rapid test) [49]; however, where testing involves an element of diagnostic infrastructure such as the GeneXpert, which, although widespread is not available in every clinic, a hub-and-spoke model may be appealing. This model of delivery of diagnostic services has been shown to be highly cost-effective in the provision of care for other infectious diseases—such as Tuberculosis [21,23] and Malaria [50], as well as HCV [51]. Demonstration of its success will add another option to facilitate treatment commencement; however, as this study has shown, careful planning and optimisation of the laboratory quality control processes are essential if this model is to be implemented successfully.

The study has several limitations. Despite the relatively small sample size, the overall sensitivity and specificity of the Xpert HCV Viral Load test for HCV RNA detection by fingerstick was excellent; however, further evaluation of this assay is crucial and should be conducted in similar settings to confirm the findings’ reproducibility. While the losses-to-follow up in the ITT population due to the events of the SARS-CoV2 pandemic and subsequent lockdowns in 2020 were unanticipated, almost all patients who commenced treatment completed their regimen (99%; 153/154). Considering the high efficacy of sofusbuvir/daclatasvir, the patients lost to follow-up would be expected to have achieved a SVR [52]. The inclusion of interventions to improve follow-up, however, would further facilitate patients achieving a SVR [40,41]. Finally, a selection bias may have contributed to the findings as the participants were already engaged in HIV care at the clinics. Reaching those at higher risk and who are less engaged with healthcare may be challenging, particularly given the recent changes in healthcare delivery in Myanmar [53]. 

## 5. Conclusions

In conclusion, the study shows excellent SVR rates among those who returned for post-treatment HCV RNA testing, and a high sensitivity and specificity of the Xpert HCV VL Fingerstick Assay for HCV RNA detection by fingerstick capillary whole-blood compared to the local gold standard method of testing in HIV/HCV co-infected people in Yangon, Myanmar. The study also demonstrated the feasibility and promise of the hub-and-spoke model in a resource-limited setting, with the microvette collection vessel addressing delays in testing and preventing a loss of samples or the opportunity to collect from patients. This highlights the feasibility of this approach, but also suggests that optimal sample handling and laboratory quality control is essential. This study highlights the potential of a simplified treatment protocol with a hub-and-spoke testing model to improve the testing, diagnosis, linkage to care and DAA therapy initiation in HIV/HCV co-infected individuals and potentially other vulnerable populations living with HCV globally.

## Figures and Tables

**Table 1:** Baseline socio-demographic and clinical characteristics.

Enrolment Characteristics	Total *n* (%) 194
Age (median, IQR)	39 (29–47)
18–29	52 (27%)
30–44	76 (39%)
45–59	59 (30%)
60+	7 (4%)
Gender	
Male	140 (72%)
Female	54 (28%)
Education	
Did not complete high school	149 (77%)
Completed high school	45 (23%)
Employment (full or part time)	
Not employed	80 (41%)
Employed	114 (59%)
Housing	
Own house	101 (52%)
Rental house	37 (19%)
Staying with family or friends	50 (26%)
Other	6 (3%)
Ever been in prison	
No	154 (79%)
Yes	40 (21%)
Current injecting drug use ^1^	
No	165 (85%)
Yes	29 (15%)
Current tobacco smoking	
No	114 (59%)
Yes	80 (41%)
Hazardous alcohol use ^2^	
No	163 (84%)
Yes	31 (16%)
CD4	
Not done	20 (10%)
<100 cells/μL	26 (14%)
100–200 cells/μL	19 (9%)
>200 cells/μL	129 (67%)
APRI score	
<2	179 (92%)
≥2	15 (8%)
Antiretroviral therapy (ART)	
No	29 (15%)
Yes	165 (85%)

^1^ Current (injected in last 30 days). ^2^ Alcohol Use Identification Test (AUDIT-c) Hazard score.

**Table 2:** Predictors of SVR12 (ITT and mITT); adjusted odds ratio presented.

	ITT				mITT			
Variables	*n* (%)	aOR	95%CI	*p* Value	*n* (%)	aOR	95%CI	*p* Value
Age (median, IQR)	39 (29–49)				40 (29;49)			
18–29	44 (29%)		reference		37 (29%)		reference	
30–44	55 (36%)	0.43	0.15–1.26	0.12	42 (33%)	0.27	0.03–2.42	0.24
45–59	49 (32%)	1.01	0.31–3.36	0.97	43 (34%)	0.22	0.01–2.83	0.25
60+	6 (4%)	2.48	0.11–56.87	0.57	6 (4%)	0.43	0.01–17.09	0.65
Gender								
Male	111 (72%)		reference		91 (71%)		reference	
Female	43 (28%)	1.43	0.48–4.28	0.51	37 (29%)	2.26	0.33–15.37	0.40
Education								
Did not complete high school	118 (77%)		reference		97 (76%)		reference	
Completed high school	36 (23%)	1.83	0.65–5.17	0.25	31 (24%)	1.94	0.27–13.70	0.50
Employment (full or part time)								
Not employed	59 (38%)		reference		46 (36%)		reference	
Employed	95 (62%)	2.79	1.15–6.79	0.02	82 (64%)	2.08	0.39–10.98	0.38
Housing								
Own house	78 (51%)		reference		63 (49%)		reference	
Rental house	32 (21%)	1.15	0.38–3.50	0.80	26 (20%)	0.32	0.03–3.46	0.35
Staying with family or friends	39 (25%)	0.94	0.34–2.57	0.90	34 (27%)	0.40	0.01–1.02	0.05
Other	5 (3%)	6.44	0.25–162.92	0.25	5 (4%)	0.18	0.01–6.00	0.34
Ever been in prison								
No	122 (79%)		reference		105 (82%)		reference	
Yes	32 (21%)	0.47	0.17–1.32	0.15	23 (18%)	1.07	0.14–7.72	0.94
Current injecting drug use ^1^								
No	144 (93%)		reference		110 (86%)		reference	
Yes	10 (7%)	0.57	0.12–2.69	0.47	18 (14%)	0.18	0.01-3.35	0.25
Current tobacco smoking								
No	86 (56%)		reference		76 (59%)		reference	
Yes	68 (44%)	0.55	0.16–1.31	0.14	52 (41%)	6.90	0.54–88.07	0.13
Hazardous alcohol use ^2^								
No	126 (82%)		reference		106 (83%)		reference	
Yes	28 (18%)	0.46	0.16–1.31	0.14	22 (17%)	0.75	0.08–6.39	0.79
CD4								
Not performed	15 (10%)	1.16	0.18–7.34	0.87	12 (9%)	1.25	0.01–173.90	0.92
<100 cells/μL	17 (11%)		reference		13 (10%)		reference	
100–200 cells/μL	13 (8%)	1.44	0.19–10.80	0.72	11 (9%)	0.61	0.01–72.74	0.84
>200 cells/μL	109 (70%)	1.13	0.29–4.34	0.85	92 (72%)	0.16	0.01–5.83	0.32
APRI score								
<2	141 (92%)		reference		121 (95%)		reference	
≥2	13 (8%)	0.26	0.07–0.88	0.03	7 (5%)	5.91	0.12–288.71	0.37
Antiretroviral therapy (ART)								
None	22 (14%)		reference		18 (14%)		reference	
On ART	132 (86%)	1.37	0.44–4.21	0.58	110 (86%)	7.16	1.03–49.50	0.04

^1^ Current (injected in last 30 days). ^2^ Alcohol Use Identification Test (AUDIT-c) Hazard score.

**Table 3:** Virological failure characteristics by participant.

Variables	1	2	3	4	5	6	7	8	9	Summary *n* (%)
Age	24	26	27	35	36	43	44	49	52	36 (27–47) ^4^
Gender	Male	Male	Male	Male	Male	Male	Female	Female	Male	7/9 (78%) male
Education (high school)	No	Yes	No	No	Yes	No	No	No	No	2/9 (22%)
Employment (full or part time)	No	No	Yes	Yes	No	No	Yes	No	Yes	4/9 (44%)
Housing	Rental house	Staying with family or friends	Staying with family or friends	Staying with family or friends	Staying with family or friends	Rental house	Staying with family or friends	Staying with family or friends	Own house	6/9 (67%) Staying with family or friends
Ever been in prison	Yes	No	Yes	Yes	Yes	Yes	No	Yes	No	6/9 (67%)
Current injecting drug use ^1^	No	Yes	Yes	No	No	Yes	No	No	No	3/9 (33%)
Current tobacco smoking	No	Yes	Yes	Yes	No	Yes	No	No	No	4/9 (44%)
Hazardous alcohol use ^2^	No	Yes	Yes	Yes	Yes	No	No	No	No	4/9 (44%)
ART	No	No	No	No	Yes	No	Yes	Yes	Yes	4/9 (44%)
CD4 (cells/ul)	216	222	420	276	342	306	221	383	529	306 (222–402) ^4^
APRI	1.33	0.13	0.43	0.70	0.81	0.13	0.38	0.32	0.33	0.38 (0.23–0.76) ^4^
Screening HCV VL ^3^	6.3	6.4	4.5	6.4	6.5	6.9	6.0	6.7	5.4	6.4 (5.8–6.6) ^4^
SVR12 HCV VL ^3^	4.9	3.6	2.5	6.2	5.1	6.4	5.6	6.6	5.4	5.4 (4.7–6.3) ^4^

^1^ Current (injected in last 30 days). ^2^ Alcohol Use Identification Test (AUDIT-c) Hazard score.^3^ log_10_ IU/mL. ^4^ median (IQR) reported.

**Table 4:** Sensitivity and specificity of the Xpert HCV VL Fingerstick Assay for HCV RNA detection compared with the standard of care.

	Quantifiable	Unquantifiable *	Total
Xpert HCV VL Fingerstick Assay (Finger-stick capillary whole blood)			
Detected	163	1	164
Undetected *	1	134	135
Total	164	135	299

Xpert HCV VL Fingerstick Assay lower limit of detection: 10 IU/mL. * HCV RNA not detectable or detectable but not quantifiable.

## Data Availability

The data presented in this study are available on request from the corresponding author. The data are not publicly available due to the sensitive nature of some of the data, including that related to injection drug use.

## Supplementary Materials

The following supporting information can be downloaded at: https://www.mdpi.com/article/10.3390/v15020521/s1, Figure S1: Study sampling profile; Table S1: EQ-5D-EL Utility test: Performance status of participants, Table S2: Sensitivity and specificity of the Xpert HCV VL Fingerstick Assay for HCV detection compared with standard of care, performed as first-pass.
