# Supplementary material for: Evaluation of Simplified HCV Diagnostics in HIV/HCV Co-Infected Patients in Myanmar
Source: Viruses. 2023 Feb 13;15(2):521. doi: 10.3390/v15020521 (PMC9967037; doi:10.3390/v15020521)
Supplement: Supplementary file 1 [file viruses-15-00521-s001.zip › Supplementary figure S1 caption.pdf]

Figure S1. Study sampling profile

205 participants were assessed for eligibility, 194/205 (95%) participants had samples available for testing, and 11/205 (5%) were excluded due to no sample available. A total of 316 finger-stick capillary whole-blood samples were collected (194 at the screening visit, 122 at SVR visit). A total of 322 standard of care samples were collected (194 at the screening visit, 128 at SVR visit). Of the 194 finger-stick capillary whole-blood samples collected at the screening visit, 20 retests were available (5 Error, 15 Invalid). After retesting 3 Error result samples were not retested, resulting 191 valid Xpert HCV Viral load results via finger-stick at the screening visit. Of the 122 finger-stick capillary whole-blood samples collected at SVR12 visit, 24 retests were available (4 Error, 20 Invalid). After retesting, 1 Error result and 13 invalid result samples were not retested, resulting in 108 valid Xpert HCV Viral load results via finger-stick at the SVR12 visit. A total of 299 valid HCV Viral load sample results via finger-stick and 322 valid standard of care results were available, and 299 results were available for both assays (final analysis population).
