# Supplementary material for: Evaluation of Simplified HCV Diagnostics in HIV/HCV Co-Infected Patients in Myanmar
Source: Viruses. 2023 Feb 13;15(2):521. doi: 10.3390/v15020521 (PMC9967037; doi:10.3390/v15020521)
Supplement: Supplementary file 1 [file viruses-15-00521-s001.zip › Supplementary table S1.pdf]

An EQ-5D-EL utility test of participants self-rated health (mobility, personal care, usual activities, pain/discomfort, anxiety/depression), and perception of overall health using a visual analogue scale (0-100). This test was completed by all 194 participants who were tested pre-treatment for HCV RNA. A median score of 85 (IQR 85;90) was reported for participant perception of overall health.

**EQ-5D-EL dimension**	*n* (%)
**Mobility**	
No problems in walking around	160 (82%)
Some problems in walking around	32 (17%)
Mostly confined to bed	2 (1%)
**Personal care**	
No problems with personal care	178 (92%)
Some problems washing or dressing myself	16 (8%)
Unable to wash or dress oneself	0 (0%)
**Usual activities**	
No problems with performing usual activities	172 (89%)
Some problems with performing usual activities	20 (10%)
Unable to perform usual activities	2 (1%)
**Pain/discomfort**	
No pain or discomfort	139 (72%)
Moderate pain or discomfort	49 (25%)
Extreme pain or discomfort	6 (3%)
**Anxiety/depression**	
Not anxious or depressed	128 (66%)
Moderately anxious or depressed	62 (32%)
Extremely anxious or depressed	4 (2%)
**Own perception of good health on visual analogue scale (1-100) (median; interquartile range [IQR])**	85 (80;90)

Table S1. EQ-5D-EL Utility test: Performance status of participants.
