# Supplementary material for: Evaluation of Simplified HCV Diagnostics in HIV/HCV Co-Infected Patients in Myanmar
Source: Viruses. 2023 Feb 13;15(2):521. doi: 10.3390/v15020521 (PMC9967037; doi:10.3390/v15020521)
Supplement: Supplementary file 1 [file viruses-15-00521-s001.zip › Supplementary Table S2.pdf]

In 272 paired samples where first pass testing (testing performed without repeat testing of *invalid* or *error* results) was performed the sensitivity of the Xpert HCV VL Fingerstick Assay was 99.3% (95% CI 96.2 – 99.9%), and specificity was 99.2% (95% CI 95.6 – 99.9). The positivity predictive value (PPV) was 99.3% (95% CI 95.4% – 99.9%) and negative predictive value (NPV) was 99.2% (95% CI 94.6% – 99.8%).

	**Quantifiable**	**Unquantifiable***	**Total**
Xpert® HCV VL Fingerstick Assay **(Finger-stick capillary whole blood)**			
**Detected**	146	1	147
**Undetected***	1	124	125
**Total**	147	125	272

HCV=hepatitis C virus.

Table S2. Sensitivity and specificity of the Xpert® HCV VL Fingerstick Assay for HCV detection compared with standard of care, performed as first-pass

Xpert® HCV VL Fingerstick Assay lower limit of detection 10 IU/mL. *HCV RNA not detectable or detectable but not quantifiable
