# Supplementary figures and images for: Evaluation of Simplified HCV Diagnostics in HIV/HCV Co-Infected Patients in Myanmar
Source: Viruses. 2023 Feb 13;15(2):521. doi: 10.3390/v15020521 (PMC9967037; doi:10.3390/v15020521)

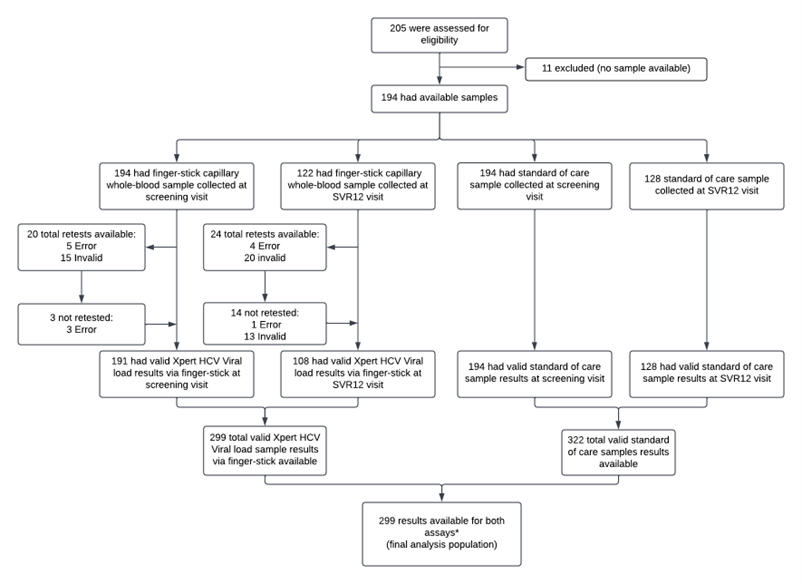

Supplement: Supplementary file 1 [file viruses-15-00521-s001.zip › Supplementary Figure S1.tif]
